# Characterization of Microbial Mat Microbiomes in the Modern Thrombolite Ecosystem of Lake Clifton, Western Australia Using Shotgun Metagenomics

**DOI:** 10.3389/fmicb.2016.01064

**Published:** 2016-07-06

**Authors:** John G. Warden, Giorgio Casaburi, Christopher R. Omelon, Philip C. Bennett, Daniel O. Breecker, Jamie S. Foster

**Affiliations:** ^1^Department of Geological Sciences, University of Texas at Austin, AustinTX, USA; ^2^Space Life Science Lab, Department of Microbiology and Cell Science, University of Florida, Merritt IslandFL, USA

**Keywords:** thrombolite, microbialite, stable isotope, Lake Clifton, microbial mat

## Abstract

Microbialite-forming communities interact with the environment and influence the precipitation of calcium carbonate through their metabolic activity. The functional genes associated with these metabolic processes and their environmental interactions are therefore critical to microbialite formation. The microbiomes associated with microbialite-forming ecosystems are just now being elucidated and the extent of shared pathways and taxa across different environments is not fully known. In this study, we profiled the microbiome of microbial communities associated with lacustrine thrombolites located in Lake Clifton, Western Australia using metagenomic sequencing and compared it to the non-lithifying mats associated with surrounding sediments to determine whether differences in the mat microbiomes, particularly with respect to metabolic pathways and environmental interactions, may potentially contribute to thrombolite formation. Additionally, we used stable isotope biosignatures to delineate the dominant metabolism associated with calcium carbonate precipitation in the thrombolite build-ups. Results indicated that the microbial community associated with the Lake Clifton thrombolites was predominantly bacterial (98.4%) with Proteobacteria, Cyanobacteria, Bacteroidetes, and Actinobacteria comprising the majority of annotated reads. Thrombolite-associated mats were enriched in photoautotrophic taxa and functional genes associated with photosynthesis. Observed δ^13^C values of thrombolite CaCO_3_ were enriched by at least 3.5‰ compared to theoretical values in equilibrium with lake water DIC, which is consistent with the occurrence of photoautotrophic activity in thrombolite-associated microbial mats. In contrast, the microbiomes of microbial communities found on the sandy non-lithifying sediments of Lake Clifton represented distinct microbial communities that varied in taxa and functional capability and were enriched in heterotrophic taxa compared to the thrombolite-associated mats. This study provides new insight into the taxa and functional capabilities that differentiate potentially lithifying mats from other non-lithifying types and suggests that thrombolites are actively accreting and growing in limited areas of Lake Clifton.

## Introduction

Microbialites are carbonate buildups that form through the interactions between microbial communities and their local environment (Burne and Moore, 1987). First appearing in the Archean (Grotzinger and Knoll, 1999), microbialites were one of the dominant ecosystems on Earth for more than 3 billion years until they declined in the Ordovician (Kennard and James, 1986). Modern analogs of these ancient ecosystems are rare compared to their abundance in the fossil record but have been found in a wide range of habitats including hypersaline (e.g., Arp et al., 1998) and freshwater lakes (e.g., Laval et al., 2000) as well as marine environments (e.g., Logan, 1961; Dravis, 1983). Microbialites can be classified based on their internal microfabrics and include the well-studied laminated stromatolites (e.g., Reid et al., 2000) and less well-known thrombolites, which are composed of unlaminated calcium carbonate mesoclots and cavities filled with detrital sediments (Kennard and James, 1986).

Currently, there are relatively few known sites of actively growing thrombolites. The most well-known are the marine thrombolites of Highborne Cay, The Bahamas (Myshrall et al., 2010; Mobberley et al., 2012, 2013, 2015) and the lacustrine thrombolites of Lake Clifton, Western Australia (Moore, 1987; Moore and Burne, 1994; Smith et al., 2010). Lake Clifton, in particular, serves as an important model system for the formation and growth of thrombolites because changes to lake water chemistry over the past 20 years. Lake Clifton has undergone a steady increase in salinity since the first published measurements in the early 1970s (Williams and Buckney, 1976). The lake was first characterized as hyposaline, with total dissolved solids (TDSs) that typically ranged between 15–32 g/L in the 1980s but by the late 1990s increased to 25–49 g/L (Moore, 1987; Rosen et al., 1996; Knott et al., 2003; Smith et al., 2010). These increases in salinity, coupled with elevations in nitrogen concentrations, have potentially altered the dominant microbial communities in the system and constrained thrombolite formation (Gleeson et al., 2015; Warden, 2016).

The recent changes in lake water chemistry observed in Lake Clifton present a unique opportunity to understand how microbialite-forming ecosystems respond and adapt to environmental changes and to understand how those changes in community composition potentially influence mat lithification. There are several key microbial metabolisms that influence the net carbonate precipitation and dissolution in microbialites including photosynthesis, heterotrophy, sulfate reduction, sulfide oxidation, and exopolymeric substance (EPS) production (Dupraz and Visscher, 2005; Visscher and Stolz, 2005; Dupraz et al., 2009). Together these metabolisms can control the precipitation potential within the microbial community by influencing the calcium carbonate saturation index and the availability of nucleation sites. Metabolisms, such as photosynthesis and some types of sulfate reduction, can increase local pH and thereby promote precipitation, whereas other metabolisms, such as sulfide oxidation and aerobic respiration, can lower the pH (Glunk et al., 2011; Gallagher et al., 2012). The availability of nucleation sites can be controlled by the production and degradation of EPSs (Dupraz et al., 2009). EPS material can bind cations, such as Ca^2+^, and through heterotrophic degradation these cations can be released and be made available for calcium carbonate precipitation (Gallagher et al., 2012).

Recently through metagenomic and metatranscriptomic analyses the genes and taxa associated with these key pathways have started to be delineated in modern microbialites (Breitbart et al., 2009; Khodadad and Foster, 2012; Mobberley et al., 2013, 2015; Saghaï et al., 2015; Casaburi et al., 2016; Ruvindy et al., 2016; White et al., 2016). These advances have increased understanding of the functional capability of microbialite-forming ecosystems and suggest that all microbialites share key pathways associated with carbonate precipitation regardless of environmental habitat, and that these metabolic pathways are more critical for microbialite development than a particular taxonomic composition (Casaburi et al., 2016).

Although these prior molecular studies have begun to characterize microbialite-forming communities in marine and hypersaline environments, there is very little information regarding the microbiomes (i.e., taxa and functional genes) of thrombolite-associated communities in Lake Clifton. One recent study targeted the 16S rRNA gene and revealed that the dominant bacterial phyla associated with thrombolite samples were Proteobacteria, Bacteroidetes, and Firmicutes (Gleeson et al., 2015). These results differ from studies conducted more than 20 years ago in which the phylum Cyanobacteria, in particular *Scytonema*, were the dominant taxa in the communities and considered critical drivers of calcification in Lake Clifton thrombolites (Moore, 1987). Recent microscopy-based studies have suggested that the composition of microbial communities in Lake Clifton has been fundamentally altered over the timespan of the observed lake water salinity increase (Smith et al., 2010). These studies have been critical to assess whether the microbial communities are changing in response to increased salinity and potential eutrophication of Lake Clifton, or if the communities are influenced by seasonal variations in lake water salinity and chemistry.

Lake Clifton is highly dynamic, with large variations in lake water salinity correlating with lake level fluctuations up to 1 m annually as the balance between water inputs and evaporative outputs changes seasonally (Moore, 1987; Rosen et al., 1996). Additionally, Lake Clifton thrombolites span well over 6 km of shoreline along which thrombolite size and morphology varies (Moore and Burne, 1994). It is unclear how microbial communities have adapted to increasing salinity across these large spatial gradients and how this has impacted thrombolite growth. Characterization of microbial communities from multiple sampling times and locations within the lake is thus essential to assess spatial and seasonal variability and fully evaluate the current potential, if any, for mat lithification in Lake Clifton. Building upon previous work, the aim of this study was to assess the functional gene capability of the microbial mats associated with the thrombolites in the lake and adjacent non-lithifying sediments to help elucidate controls on carbonate lithification potential.

In this study, we characterized the taxonomic composition and functional gene complexity of microbial mats across a 13 km transect along the eastern foreshore of Lake Clifton. We compared microbial mats associated with thrombolites to those mats forming on sandy lakebed sediments to determine whether differences in the mat microbiomes, particularly with respect to metabolic pathways and environmental interactions, may potentially contribute to thrombolite formation. Stable isotope biosignatures were used to delineate the dominant metabolism(s) associated with calcium carbonate precipitation in the thrombolite build-ups. This work provides new information regarding the current composition of microbial mat communities in Lake Clifton and serves as a baseline for future work examining the effect of the continued salinity increase in the lake. Additionally, by targeting a lacustrine site that has experienced distinctive environmental shifts over the past few decades, these results expand our understanding of the modern microbialite microbiome and help delineate those core pathways and metabolisms essential for microbialite formation.

## Materials and Methods

### Sample Collection

**FIGURE 1:**
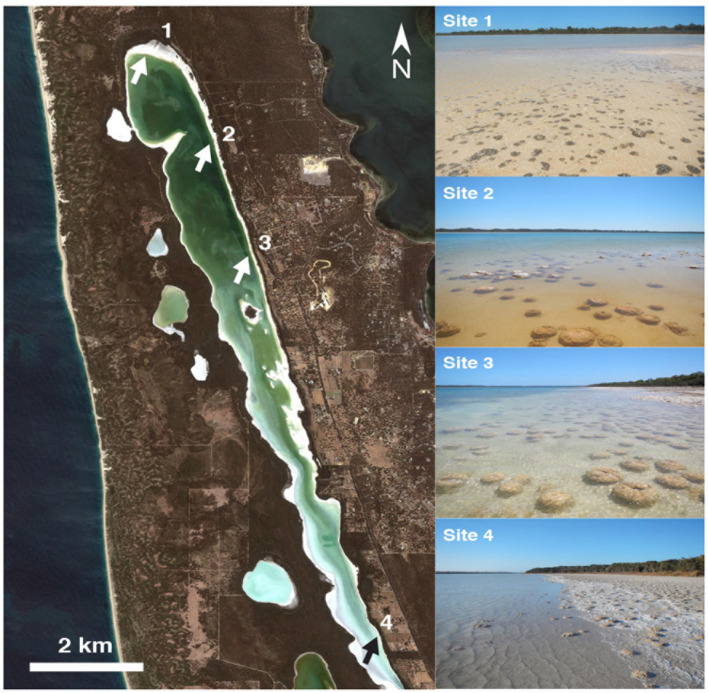
**Map of study area and photographs of sampling sites.** Thrombolite-associated mats were observed and sampled from Sites 1 and 2. Sediment-associated mats were observed throughout the lake and sampled from Sites 2, 3, and 4. Aerial image from Google Earth using satellite data collected on February 12, 2014.

A survey was conducted in February 2014 to identify the predominant microbial mat communities present in Lake Clifton. For clarity, we define thrombolite-associated mats as microbial mats growing on lithified thrombolite structures and sediment-associated mats as microbial mats growing on sandy non-microbialite forming lakebed sediments. This survey spanned ∼13 and ∼2 km along the eastern and western shorelines, respectively, extending southward from the northern most portion of the lake. A total of six microbial mat samples were collected from four research sites chosen based on survey results (**Figure 1**). Samples were collected in triplicate using a Harris 8.0 mm Uni-Core sampler (Ted Pella, Inc., Redding, CA, USA) and immediately immersed and stored in RNAlater until processing (Life Technologies, Inc., Grand Island, NY, USA). Of the six total mat samples, three were obtained from thrombolite-associated mats and three were obtained from sediment-associated mats. The mat samples were collected in February 2014 under license number SF009605 from the Western Australia Department of Parks and Wildlife. The pH and specific conductance of lake water at each research site was measured using an Ultrameter II (Myron L Company, Carlsbad, CA, USA) calibrated to 4, 7, and 10 pH buffer standards and high conductivity NaCl standards (58670 and 111900 μS/cm).

### DNA Extraction and Sequencing

Genomic DNA was extracted from microbial mat samples using a modified xanthogenate bead beating method as described previously (Foster et al., 2009; Khodadad and Foster, 2012). Each sampling replicate was homogenized and underwent multiple extractions (between 2 and 5 extractions per replicate) to accumulate enough high quality DNA for sequencing. The recovered DNA was then pooled into a single sample representing each mat type. DNA was sequenced on an Illumina HiSeq2000 (100-bp non-overlapping, paired-end reads) platform at MR DNA (Shallowater, TX, USA). All sequences have been deposited in the NCBI sequencing read archive (SRP072185) under project number PRJNA315989.

### Taxonomic and Functional Annotation of Metagenomes

Metagenomic sequences were quality filtered and trimmed using sickle version 1.33 (Joshi and Fass, 2011) following default usage for paired-end reads. Trimmed unassembled reads were annotated using Metagenome Composition Vector (MetaCV) version 0.230 (Liu et al., 2013) with default parameters and a reference database of 2,059 genomes and 6,553,878 genes provided by MetaCV. MetaCV classifies reads into taxonomic and functional groups using a composition and phylogeny-based approach. Additionally, MetaCV is alignment-based and an assembly based approach was not used because of several limitations when analyzing microbial communities of complex systems. These include but are not limited to: poor quality assembly for less represented species, masking of actual taxonomic depth, and chimeric assembly events especially for closely related species (Liu et al., 2013; Howe and Chain, 2015; Casaburi et al., 2016). Correlation scores were determined for each read according to the confidence of the classification by MetaCV. Reads having a correlation score of less than 20 were discarded following the recommendation of MetaCV developers (Liu et al., 2013) for 100 bp read lengths and following cutoff values used in similar studies (Deusch et al., 2014). MetaCV generates summaries of taxonomic classifications to the deepest reachable rank and multiple functional classifications, including KEGG, COG, and eggNOG databases (Kanehisa and Goto, 2000; Tatusov et al., 2003; Muller et al., 2010). This analysis focused on KEGG orthology classifications. Taxonomic data were plotted using Krona (Ondov et al., 2011) and for visualization purposes only the most abundant taxa (>0.5%) are shown.

### Visualization and Statistical Analyses

Exploratory and differential abundance analyses were performed in R 3.2.2 and MEGAN 5.10.5 (Huson et al., 2011). Microbial diversity analyses were performed using raw counts normalized by a sequencing depth of 221,168 sequences/sample. Rarefaction curves were computed using the number of observed species while Bray–Curtis distances were plotted as principal coordinate analysis (PCoA) in MEGAN. Agglomerative clustering analyses were performed with Bray–Curtis distances as input and the UPGMA clustering method specified (Bray and Curtis, 1957). The statistical significance of diversity analyses was assessed by PERMANOVA using the adonis function in the vegan R package (Oksanen et al., 2011). Statistically significant differences in non-normalized taxonomic and functional classifications among thrombolite- and sediment-associated microbial mats were identified using negative binomial Wald tests as implemented in DESeq2 (Love et al., 2014). Raw *p*-values were corrected for multiple testing using the Benjamini–Hochberg adjustment and adjusted *p*-values < 0.05 were considered to indicate differentially abundant classifications between thrombolite- and sediment-associated microbial mats.

### Stable Isotope Measurements

Vertical core samples were collected from two thrombolites using a serrated 4 cm diameter stainless steel corer and used to measure δ^13^C values of calcium carbonate in the thrombolites. Thrombolite material was subsampled from each core in 5 cm intervals from 0 to 40 cm depth. Each sample was ground into a powder using a mortar and pestle and reacted with hydrogen peroxide (30%) to remove organic material. Approximately 400 μg of each subsample were transferred into septum-capped Labco^®^ exetainer vials, which were then capped and flushed with ultra-high purity helium. Phosphoric acid (103%) was then injected to liberate CO_2_ and analyzed using a Thermo Scientific GasBench II coupled to a Thermo Scientific MAT 253 Stable Isotope Ratio Mass Spectrometer operating in continuous flow mode (Spötl and Vennenmann, 2003). NBS-18 and NBS-19 calcite standards were analyzed alongside unknowns. The external reproducibility of an internal laboratory calcite standard measuring 4.44‰ was ± 0.01‰ (1σ).

Water samples were collected from Lake Clifton and from private wells located inland of the lake for measurement of dissolved inorganic carbonate (DIC) δ^13^C values. The temperature of water samples was measured during sample collection. Water samples were filtered to 0.22 μm into serum bottles without headspace and crimp-sealed with butyl rubber septum stoppers. In the laboratory, one-milliliter aliquots were transferred from sample bottles to septum-capped exetainers previously flushed with He. Phosphoric acid (103%) was added to drive DIC into the headspace as CO_2_. This acidification was allowed to proceed for 12 h at 40°C after which δ^13^C values of headspace CO_2_ were measured using a Thermo Scientific GasBench II coupled to a Thermo Scientific MAT 253 Stable Isotope Ratio Mass Spectrometer operating in continuous flow mode (Assayag et al., 2006). A sodium bicarbonate internal laboratory standard that was dissolved in deionized water was analyzed alongside unknowns. The δ^13^C value of internal laboratory standard was calibrated against NBS-18 and NBS-19 calcite standards and measured -19.44‰. The external reproducibility of replicate analyses of the laboratory standard was ±0.03‰ (1σ). Stable carbon isotope data reported in permil (‰) were normalized to the PDB scale by assigning NBS-19 a δ^13^C value of 1.95‰ (Coplen, 1996).

## Results and Discussion

### Lake Clifton Site Description and Overview

Thrombolites have been shown to be located primarily on the eastern foreshore of Lake Clifton and occur both as isolated structures (<1.3 m height) and as part of a reef complex that spans more than 6 km of the shoreline (**Figure 1**; Moore and Burne, 1994). To assess the current extent of actively forming thrombolites, if any, in the lake, a survey was conducted in February 2014 to identify locations where microbial mats were found growing in association with thrombolite build-ups. Four research sites were chosen that represented the end members of the different mat types and growth characteristics observed during the survey (**Figure 1**; **Table 1**). Lake water pH at the different research sites ranged from 7.18 to 7.63 and specific conductance ranged from 121.4 to 188.2 mS/cm, which results in an estimated salinity range of 86.3–139.1 g/L according to conductivity-salinity regression for Australian salt lakes in Williams (1986).

**Table 1:** Sample collection metadata.

Sample ID	Type	Site	SC (mS/cm)^a^	pH^b^	Description
LCT14M-09	Thrombolite	1	130.2	7.63	Dark green microbial mat growing on small thrombolites (<20 cm diameter)
LCT14M-12	Thrombolite	2	121.4	7.54	Green EPS-rich mat growing on thrombolites
LCT14M-14	Thrombolite	2	121.4	7.54	Desiccated green EPS-rich mat growing on thrombolites
LCT14M-11	Sediment	2	121.4	7.54	Loosely cohesive microbial mat growing on sediment
LCT14M-22	Sediment	3	124.8	7.54	Loosely cohesive microbial mat growing on sediment
LCT14M-21	Sediment	4	188.2	7.18	Cohesive microbial mat growing on sediment, gray in color


*^a^Specific conductance (SC) of lake water at sampling site. For comparison, Indian Ocean SC measured 55.82 mS/cm.*

*^b^pH of lake water at sampling site.*

**FIGURE 2:**
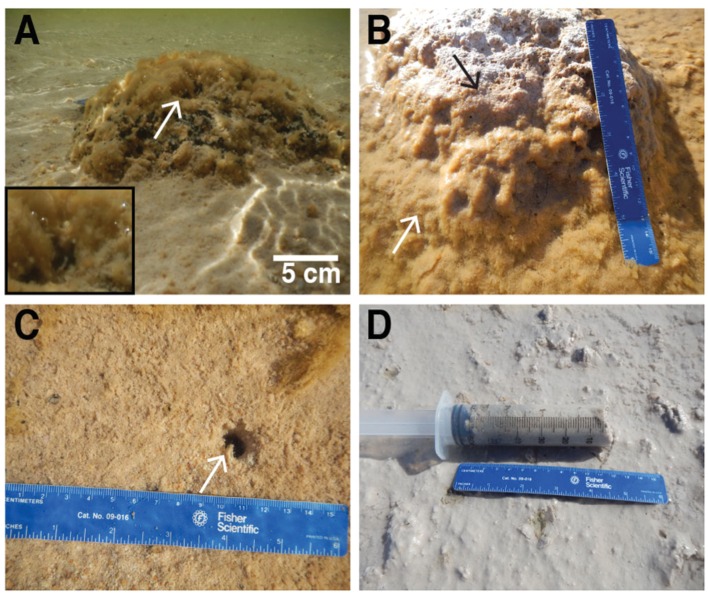
**Microbial mats in Lake Clifton, Western Australia.** **(A)** Thrombolite-associated microbial mat with oxygen bubbles (inset box shows higher magnification) observed emanating from the mat surface. White arrow denotes lower left corner of inset box. **(B)** Thrombolite-associated microbial mat (white arrow) grades upward into a desiccated mat (black arrow) consistent in appearance with lithified thrombolites. **(C)** Sediment-associated microbial mat forming on sandy lakebed sediments. **(D)** Sediment-associated microbial mat with a strong sulfide smell observed during sampling. Length of blue ruler = 16 cm. Arrows are also representative of sampling locations.

Thrombolite-associated mats were found only on a ∼2 km span of the northeastern foreshore, which was located between and included Sites 1 and 2 (**Figure 1**). At Site 1, dark green-pigmented microbial mats were observed on the surfaces of small (<15 cm diameter) isolated thrombolites (**Figure 2A**). At Site 2, located near the maximum extent of the thrombolite reef complex in Lake Clifton, thick green-pigmented microbial mats formed on submerged thrombolites (**Figure 2B**). During fieldwork, falling lake levels exposed some of these microbial mats to the atmosphere and the mats quickly became desiccated (**Figure 2B**). Thrombolite-associated microbial mats at Sites 1 and 2 were rich in EPSs and bubbles observed emanating from the mats (**Figure 2A**, inset) were determined to be oxygen using a portable dissolved oxygen meter. Thrombolites at Site 3 showed no visual evidence of microbial mat formation but loosely cohesive mats were observed on sandy lakebed sediments (**Figure 2C**). Between Sites 3 and 4 the thrombolite reef complex gradually disappears, leaving areas of isolated thrombolites (generally < 30 cm diameter) that were also absent of microbial mats. Sediment-associated mats at Site 4 were cohesive, gray in color (**Figure 2D**), and a strong sulfide smell emanated from the mat during sampling. Thrombolite-associated mat samples (*n* = 3) were collected from Sites 1 and 2 whereas sediment-associated mat samples (*n* = 3) were each collected from Sites 2, 3, and 4. The restriction of thrombolite-associated mats to Sites 1 and 2 suggests that thrombolites outside this area were inactive at the time of sampling.

### Taxonomic Composition of Thrombolite- and Sediment-Associated Mats

Metagenomic libraries were generated in triplicate using whole genome shotgun sequencing of both Lake Clifton thrombolite- (Sites 1, 2) and sediment-associated (Sites 2, 3, 4) microbial mats. The complete dataset of six libraries consisted of 71,376,595 sets of paired-end raw reads of which 2.77% were discarded during quality filtering. A summary of the reads annotated to each taxonomic level and KEGG ortholog group is provided in **Supplementary Table S1** and estimated species richness is depicted as rarefaction curves in **Supplementary Figure S1**.

A total of 33 phyla, 54 classes, and 117 orders were recovered from the metagenomes of the Lake Clifton microbial mats and results indicated they were predominantly bacterial (>97.5%) with a relatively minor proportion of Archaea (<2.5%). This result is consistent with other studies in which low diversity and relative abundance of Archaea (1.4%) was observed in both Lake Clifton (Gleeson et al., 2015) and Bahamian thrombolite-forming mats (Mobberley et al., 2013, 2015). This result, however, contrasts with weakly lithifying microbial mats from Shark Bay, Western Australia, which contained a relatively large proportion of predominantly halophilic Archaea (Ruvindy et al., 2016).

**FIGURE 3:**
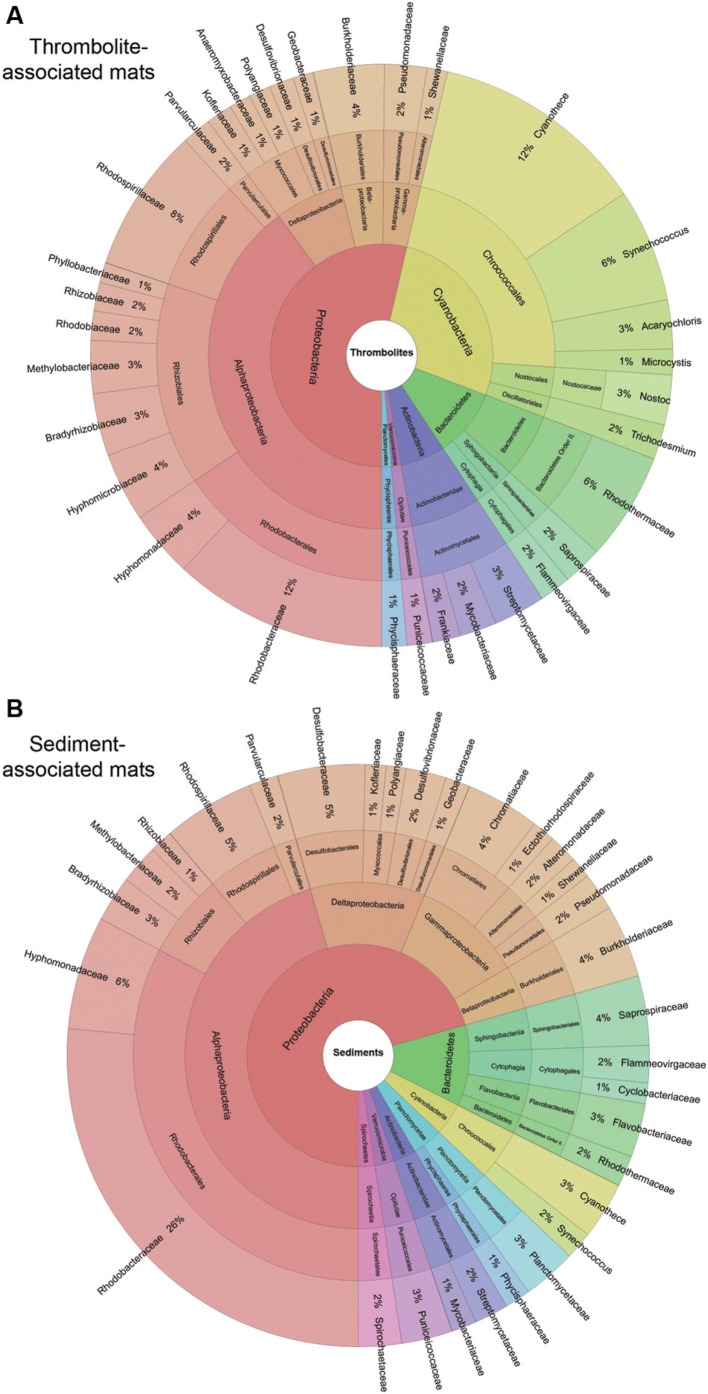
**Taxonomic composition of thrombolite- and sediment-associated mat samples.** Krona plot visualizing taxonomic hierarchies of the average community composition of **(A)** thrombolite- and **(B)** sediment-associated microbial mats. Only taxa with relative abundances greater than 0.5% are reported. Taxa are plotted on the same color scale in **(A,B)**.

The metagenomes of the thrombolite- and sediment-associated mats in Lake Clifton were diverse and exhibited several shared taxa, as depicted in **Figure 3**. Please note the values reported here reflect the percentages of the total community. Of the 33 phyla recovered from the Lake Clifton mats there were five that dominated, including: Proteobacteria (49.2–62.2%); Cyanobacteria (4.2–23.1%); Bacteroidetes (9.2–15.8%); and Actinobacteria (4.3–9.8%). The prevalence and relative abundances of these phyla in the Lake Clifton microbial mats are consistent with many lithifying and non-lithifying mat systems including hypersaline environments of Shark Bay (Ruvindy et al., 2016; Suosaari et al., 2016), marine waters of Exuma Sound, The Bahamas (Myshrall et al., 2010; Khodadad and Foster, 2012; Mobberley et al., 2013; Casaburi et al., 2016) and freshwater systems in Mexico (Breitbart et al., 2009; Couradeau et al., 2011; Nitti et al., 2012; Saghaï et al., 2015). Within the Proteobacteria, the Alphaproteobacteria (34.7–43.5%) were the most dominant class, whereas Gammaproteobacteria (11.0–14.5%), Deltaproteobacteria (5.2–10.5%), and Betaproteobacteria (4.0–7.2%) were also were represented in the mats. The most abundant Alphaproteobacteria were Rhodobacterales (8.8–23.7%), Rhizobiales (5.6–12.0%), and Rhodospirillales (2.6–6.2%) and were found in both the thrombolite- and sediment associated microbialites (**Figure 2**). These alphaproteobacterial orders are common to most lithifying and non-lithifying microbial mats (Kunin et al., 2008; Foster and Green, 2011; Khodadad and Foster, 2012; White et al., 2015; Casaburi et al., 2016; White et al., 2016). A complete summary of the taxonomic annotations is listed in **Supplementary Table S2**.

**FIGURE 4:**
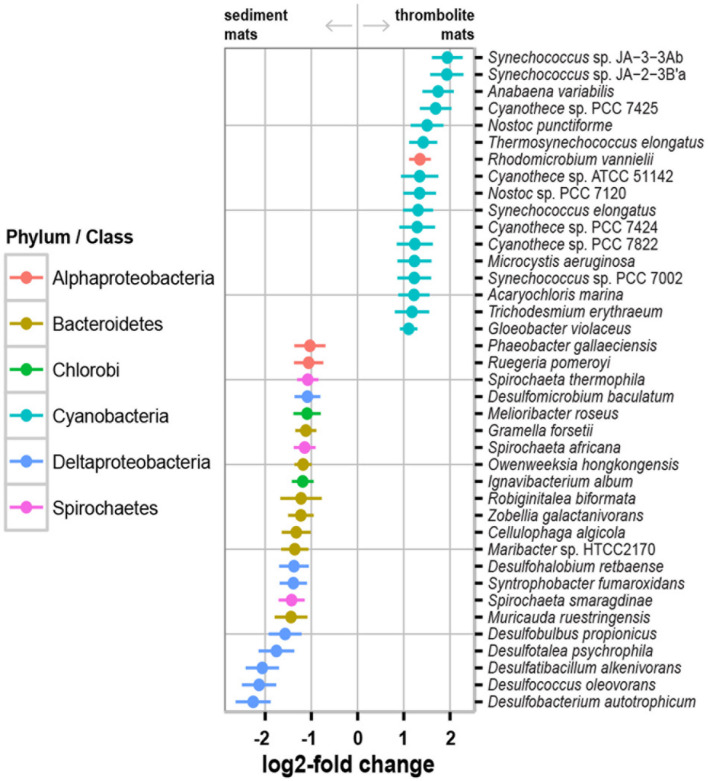
**Selected enriched taxa in thrombolite- and sediment-associated microbial mats.** A positive log2-fold change indicates taxa significantly enriched (Wald test, *p* < 0.05) in thrombolite-associated mats, whereas a negative log2-fold change indicates taxa significantly enriched in sediment-associated mats. Colors indicate the phylum or class of each data point and lines represent standard error.

Of the five dominant phyla Cyanobacteria were most enriched in thrombolite-associated mats (9.5–23.1%) compared to the sediment-associated mats (4.6–4.7%). The difference in cyanobacterial abundance was attributed to an increase in recovered reads associated with the orders Chroococcales (7.6–17.3% thrombolitic mats; 2.9–3.5% sedimentary mats) and Nostocales (1.2–3.9% thrombolitic mats; 0.69–0.74% sedimentary mats). These differences in cyanobacterial abundance were also observed at the species-level, as determined by the DESeq2 statistical package in R (Love et al., 2014). Although, the DESeq2 package was originally developed for testing differential gene expression across sample groups its statistical framework it is now being applied to metagenomic data (McMurdie and Holmes, 2014; Rodriguez-R et al., 2015). Analysis of the Lake Clifton metagenomes revealed a total of 1219 taxa annotated to the species-level. Of these species, 229 were statistically different between the thrombolite- and sediment-associated mats (Wald test, *p*-value ≤ 0.05; **Supplementary Table S3**). Due to the high number of statistically significant results a more stringent set of criteria were used to identify the most prominent differences between thrombolite- and sediment-associated microbial mat communities: (1) the absolute log2-fold change was greater than 1 (twofold difference in abundance) and (2) the average abundance (baseMean) was >2000. Filtering by these criteria resulted in 39 statistically significant species that were distinct between the two mat types (**Figure 4**). A positive log2-fold change indicates taxa significantly enriched (Wald test, *p* < 0.05) in thrombolite-associated mats, whereas a negative log2-fold change indicates taxa significantly enriched in sediment-associated mats. Of the species enriched in the thrombolite-associated mats most (*n* = 16) were cyanobacterial, belonging to the orders Chroococcales, Nostocales, Gloeobacteriales, and Oscillatoriales (Wald test, *p*-value < 0.05). All species showing statistically significant differences between the two mat types are listed in **Supplementary Table S3**. These results are consistent with the microbiomes of several other lithifying mat systems, as both Chroococcales and Nostocales orders are enriched in a wide range of habitats including Shark Bay (Suosaari et al., 2016), Exuma Sound, The Bahamas (Stolz et al., 2009; Khodadad and Foster, 2012; Mobberley et al., 2013; Casaburi et al., 2016) and the fresh water microbialites of Cuatros Ciénegas, Mexico (Breitbart et al., 2009). Although numerous Nostocales were recovered from the thrombolite-associated mats, no *Scytonema*-like species were recovered as previously reported by Moore (1987). These findings could be the result of increased salinity, as several hypersaline lithifying mat systems are rich in coccoid cyanobacteria (e.g., Suosaari et al., 2016). Concurrently, it may reflect the under-representation of cyanobacterial genomes in the MetaCV reference database and indicates the need to expand current sequencing efforts of cyanobacterial species (e.g., Saw et al., 2013).

In contrast, the non-lithifying sediment-associated mats were highly enriched in Bacteroidetes (13.0–15.8%) and Deltaproteobacteria (6.6–10.5%) when compared to the thrombolite-associated mats (9.2–9.8%; 5.2–6.6%, respectively). Of the Bacteroidetes, there was a higher representation of Flavobacteriales (4.1–9.0%) compared to thrombolite-associated mats (2.3–2.7%), specifically an enrichment of two species from the genus *Cellulophaga* and *Muricauda ruestringensis*, known to be associated with marine sediments (Bruns et al., 2001; Pati et al., 2011; **Supplementary Table S3**). Additionally, Sphingobacteriales (2.8–3.4%) were enriched in the sediment-forming mats compared to those associated with thrombolites (1.2–2.7%). High levels of Bacteroidetes have been observed in a wide range of habitats, such as hypersaline, marine and freshwater microbial mats (e.g., Wong et al., 2015; Casaburi et al., 2016; White et al., 2016) soils (e.g., Lauber et al., 2009), and the gastrointestinal tract of animals (Ley et al., 2006; Thomas et al., 2011). Members of this phylum are known for their ability to uptake and degrade a wide range of high molecular weight biopolymers (Kirchman, 2002; Church, 2008). Within the microbial mats these taxa may be playing a key role in the mineralization of high molecular weight organic matter into forms then accessible by other members of the microbial mat community.

Interestingly, sediment-associated microbial mats were also differentially enriched in Deltaproteobacteria, specifically the order Desulfobacterales (1.2–4.8%) compared to thrombolite-associated mats (0.4–0.9%). DESeq2 analyses revealed several enriched sulfate-reducing bacteria including taxa with high sequence similarity to *Desulfobulbus propionicus, Desulfotalea psychrophila, Desulfatibacillum alkenivorans, Desulfococcus oleovorans*, and *Desulfobacterium autotrophicum* (**Supplementary Table S3**). Sulfate reducing bacteria are major contributors to carbon oxidation in both microbial mats and sediments (Canfield and Des Marais, 1993) and they have been also implicated as having a major role in biologically induced carbonate precipitation by driving the alkalinity engine (i.e., the mineral precipitation potential) toward a higher saturation index (e.g., Dupraz et al., 2009; Gallagher et al., 2012).

### Functional Capability of Thrombolite- and Sediment-Associated Microbial Mat Communities

The metagenomes recovered from the Lake Clifton microbial mats contained a combined 6792 KEGG functions (KEGG level 4), which mapped to 306 KEGG level 3 pathways (**Supplementary Table S4**). Metabolisms, such as carbohydrate synthesis, photosynthesis, methane and nitrogen metabolism, accounted for 46.5–47.9% of assigned reads, whereas genetic information processing (19.9–20.9%) and environmental information processing (10.1–11.1%) were the next most abundant groupings (**Supplementary Table S4**). Approximately 16% of the total reads were unable to be classified for metabolic function. Of the 306 level 3 KEGG pathways identified in the mats, 63 dominant pathways were identified that occurred at a relative abundance greater than 0.5% and these pathways never varied more than 0.5% between sample metagenomes (**Supplementary Figure S2**).

**FIGURE 5:**
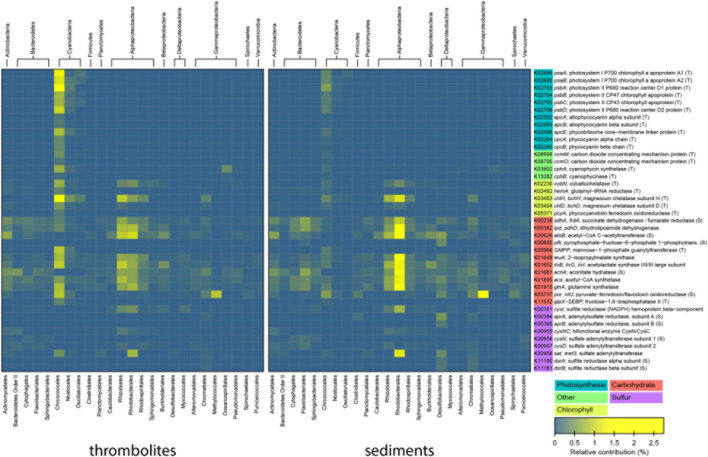
**Bacterial taxa associated with selected metabolic pathways.** Heatmap showing the relative contribution of each taxon to the total number of selected genes for thrombolite- and sediment-associated mats. The most abundant genes belonging to photosynthesis, carbohydrate, methane, and sulfur metabolisms are shown for the top 20 taxa in either mat types containing the selected genes.

The taxa associated with highly represented genes within the microbial mat metagenomes were identified by matching taxonomic information to KEGG level 4 functions in MetaCV. A heat map correlating the relative abundance of each taxon to the specific genes is shown in **Figure 5**. Within the recovered metagenomes the largest difference between the thrombolite- and sediment-associated mats were genes attributed to photosynthesis, specifically in photosystems I and II (**Figure 5**; **Supplementary Figure S3**). Most of these recovered photosynthesis genes were assigned to the order Chroococcales, and to a lesser extent the Nostocales and Oscillatoriales. Additionally, there was a significant enrichment of genes associated with photosynthesis antenna production, phycobilisome and cyanobacterial tetrapyrrole biosynthesis (chlorophyll, heme, and bilins) in the thrombolite-associated mats compared to the sediment-associated mats (**Figure 5**; **Supplementary Table S3**). Some photosynthesis-associated genes were recovered and attributed to phototrophic Alphaproteobacteria, but these were not significantly different between the mat types. Photosynthesis has been shown to be a major driver of biological-induced carbonate precipitation in lithifying microbial mat systems (Dupraz and Visscher, 2005; Dupraz et al., 2009) and can directly influence the precipitation potential of calcium carbonate by increasing pH and shifting DIC speciation toward carbonate ions (Visscher and Stolz, 2005; Riding, 2011). Recent sequencing of the metagenomes of several microbialite systems have shown that genes associated with photosynthesis are enriched in microbial mats from a diverse range of habitats including freshwater (Breitbart et al., 2009; White et al., 2016), marine (Khodadad and Foster, 2012; Mobberley et al., 2013; Saghaï et al., 2015), and hypersaline (Ruvindy et al., 2016) systems. Together, these many studies indicate that photosynthesis is a critical metabolism in the microbiome of modern microbialite-forming mats.

Although photosynthetic uptake of inorganic carbon can directly influence carbonate precipitation by raising the pH of the local environment within the mat, the transport of bicarbonate (HCO_3_^-^) into cells during carbon fixation can also favor carbonate precipitation through export of hydroxyl (OH^-^) ions outside the cell, thus increasing pH and generating alkalinity (e.g., Riding, 2011). In the thrombolite-associated mats there was a significant increase in the number of represented genes encoding carbon-concentration mechanism (CCM) proteins (e.g., ccmK, ccmL, ccmM, ccmO), bicarbonate transporters (e.g., cmpA, cmpD) and cyanophycin biosynthesis (e.g., cphA, cphB) compared to the sediment-forming mats (*p* < 0.001; **Supplementary Table S3**). Cyanophycin has long been known to be an important mechanism for nitrogen storage in cyanobacteria (Obst and Steinbüchel, 2006), but recent studies of non-heterocystous cyanobacteria derived from stromatolite-forming microbial mats of Yellowstone National Park have shown that cyanophycin plays an important role in carbon storage under low or variable carbon:oxygen ratios (Liang et al., 2014). Together, the increased number of genes encoding for CCM mechanisms and bicarbonate transporters suggest that the thrombolite-associated mats have the necessary machinery to increase the localized pH in the mats; thereby promoting favorable conditions for carbonate precipitation.

Another highly represented metabolic subsystem in both mat types was carbohydrate synthesis; however, the most abundant genes associated with this KO grouping were associated with housekeeping metabolisms, such as the citric acid cycle, Calvin cycle, and electron transport mechanisms (**Figure 5**; **Supplementary Table S4**). Interestingly, there were no extensive differences observed in the relative abundance of genes associated with the synthesis and degradation of sugars, suggesting that these metabolisms are important in both the thrombolite- and sediment- associated microbial mat communities. However, differences did occur in the relative abundances of the taxa associated with these metabolisms (**Figure 5**). In the thrombolite-associated mats the cyanobacterial order Chroococcales and alphaproteobacterial orders Rhizobiales and Rhodobacterales had the highest representation of genes associated with carbohydrate synthesis and degradation, whereas there was an enrichment of Rhodobacterales in the sediment-associated mats (**Figure 5**; **Supplementary Tables S3** and **S4**). Future studies of the Lake Clifton system would require analysis of the expression or activity of these genes (e.g., RNA- and Ribo-seq) to elucidate differences between the two mat types. A previous study comparing lithifying and non-lithifying mats showed a propensity of lithifying mats to utilize hexose sugars (e.g., mannose, galactose) and dicarboxylic acids compared to non-lithifying mats (Khodadad and Foster, 2012), which may facilitate the heterotrophic degradation of EPSs in lithifying microbial mats and induce carbonate precipitation by releasing Ca^2+^ from EPS into solution.

Although, sulfur metabolism was not highly represented in any of the Lake Clifton mat metagenomes and comprised only ∼0.4% of annotated reads, both mats harbored genes associated with both sulfur assimilation and dissimilation. However, there was a significant increase in the representation of dissimilatory sulfate reduction genes attributed to Deltaproteobacteria, including genes for, adenylyl-sulfate reductase (aprB) and dissimilatory sulfite reductase (dsrA) in the sediment-associated mats (*p* ≤ 0.001; **Figure 5**; **Supplementary Table S3**). The disproportionate increase in sulfate reducing bacteria and dissimilatory sulfate reduction in the sediment-associated mats could be related to slower diffusion of oxygen into the sediment-associated mats compared to the thrombolite-associated mats. Alternatively, the difference may reflect the particle size of the sediments, as nutrient uptake has been shown to be higher in those environments with smaller particle size (Fukui and Takii, 1996).

Another major category of genes represented within both Lake Clifton mat types was environmental response and processing genes, primarily associated with ABC transporters and two-component signaling systems (**Supplementary Figures S4** and **S5**). Genes associated with osmotic stress (envZ, ompR) and nutrient limitation (phosphate, phoR, phoB, phoP; nitrogen, glnL, glnG, ntrY, ntrX) were highly represented within both mat types, although there were distinct differences in the taxa associated with these genes (**Supplementary Figure S4**). For example, in the phosphate transport genes there was an enrichment of genes associated with the cyanobacteria, primarily the Chroococcales, in the thrombolite-associated mats, whereas in the sediment-associated mats the phosphate transport genes were primarily associated with the alphaproteobacterial orders Rhodobacteriales and Rhizobiales (**Supplementary Figure S5**). These taxonomic-based differences may reflect specific responses to N and P influx into Lake Clifton and may provide insight into how these environmental factors influence the community composition (Loza et al., 2014).

Within two-component systems, genes from the OmpR family associated with high-intensity light and nutrient stress were significantly enriched in the thrombolite-associated mats (**Supplementary Table S3**). For example, the enriched genes nblS and nblR have been shown to regulate photosynthetic machinery in response to high-intensity light and nutrient stress and are critical for cyanobacterial survival under such conditions (Schwarz and Grossman, 1998; van Waasbergen et al., 2002). The thrombolite-associated mats also showed an increase in the relative number of genes associated with ABC transporters (mntABC) that control Mn^2+^ uptake into cells. Mn is an essential component of photosynthetic machinery and when Mn is limiting, the genes manS and manR play an important role in Mn^2+^ homeostasis (Ogawa et al., 2002; Yamaguchi et al., 2002). Genes associated with Mn^2+^ homeostasis and uptake (mntB, mntC) were also enriched in thrombolite-associated mats (**Supplementary Table S3**).

**FIGURE 6:**
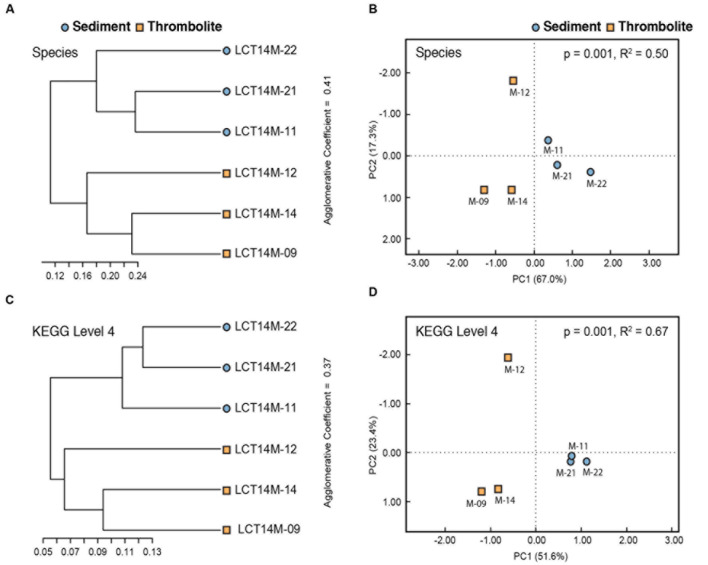
**Beta diversity analyses of species level taxonomic data and level 4 KEGG functional data.** Hierarchical clustering of Bray–Curtis distance matrices suggest that samples cluster according to mat type based on taxonomic **(A)** and functional content **(C)**. Principal coordinate analysis (PCoA) plotting Bray–Curtis distance matrices calculated from taxonomic **(B)** and functional content **(D)** also suggest clustering of samples according to mat type. PERMANOVA (adonis) indicates that grouping of samples according to mat type accounts for 50% (**B**, *p* = 0.001) of the variation in Bray–Curtis distances based on species level taxonomic data and 67% (**C**, *p* = 0.001) of variation based on level 4 KEGG functions.

In addition to profiling the specific functional pathways, a beta diversity analysis of the six metagenomes was conducted using hierarchical clustering and PCoA of Bray–Curtis distance matrices (**Figure 6**). The comparisons revealed that the thrombolite- and sediment-associated mats are distinctive communities at both the taxonomic (species) and functional (KEGG 4) level. The PCoA showed a high level of variance according to both taxonomy (PC1 67.0%; PC2 17.3%) and function (PC1 51.6%; PC2 23.4%). The type of microbial mat accounted for 50% of the taxonomic variation (adonis, *p* = 0.001) and 67% of functional gene variation (adonis, *p* = 0.001). Together, these results suggest that the microbial mats associated with the thrombolites are significantly different from those mats growing on the surrounding lake bed sediments.

The variations observed in taxonomic composition and functional capability may reflect differences in microdistribution and substrate availability between the two mat types. The nutrient flux to microbial mats growing in Lake Clifton is controlled partly by heterogeneities in groundwater flow patterns beneath thrombolites and sand-sized sediments adjacent to the thrombolites. Hydrologic observations made at Site 2, conducted concurrently with microbial sampling reported in this study, showed that groundwater springs emerge directly through thrombolites (Warden, 2016). Spring water upwelling through the thrombolites was of much lower salinity compared to adjacent sediments at similar elevations and the large groundwater flow rate through the springs resulted in phosphorus fluxes several orders of magnitude higher in the thrombolites than sediments next to the thrombolites (Warden, 2016). The differences in the salinity of the local microbial mat environments could be driving differences in the taxonomic distribution, as similar trends have been seen in stromatolite ecosystems (Casaburi et al., 2016; Suosaari et al., 2016).

### Correlating Metabolic Activity to Biologically Induced CaCO_3_ Precipitation

Resolving the relative contributions of photoautotrophic and heterotrophic metabolisms to calcium carbonate precipitation in modern microbialites is critical to understanding their formation and interpreting ancient microbialites. Biosignatures can impart a record of the metabolic processes that induce carbonate precipitation in microbialites and are often recorded by the stable carbon isotope composition of the precipitated calcium carbonate (Burne and Moore, 1987; Léveillé et al., 2007; Breitbart et al., 2009; Planavsky et al., 2009; Brady et al., 2010; Nitti et al., 2012). These biosignatures occur when metabolic activity influences the stable carbon isotope composition of the local DIC reservoir from which carbonate precipitates, resulting in measured carbonate δ^13^C values that deviate from theoretical equilibrium values. Photoautrophs preferentially use ^12^C; leaving the local DIC reservoir and precipitated carbonate enriched in ^13^C compared to theoretical values in equilibrium with bulk DIC δ^13^C measurements (e.g., Burne and Moore, 1987; Brady et al., 2010). Heterotrophic breakdown of organic matter adds ^12^C to the local DIC reservoir, leaving the local DIC reservoir and precipitated carbonate depleted in ^13^C compared to equilibrium with bulk DIC δ^13^C measurements (e.g., Andres et al., 2006; Breitbart et al., 2009).

**FIGURE 7:**
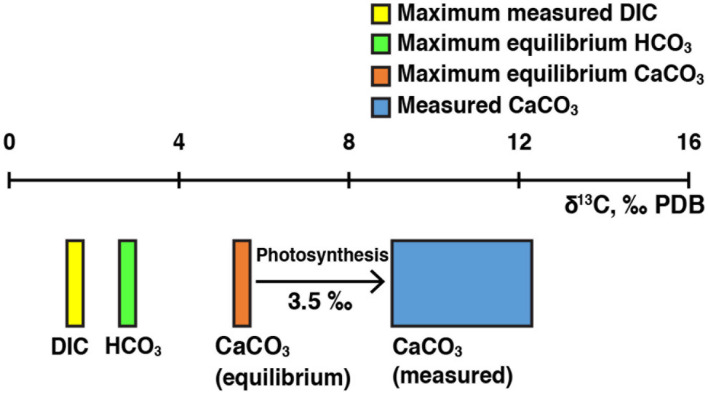
**Photosynthetic biosignatures in thrombolitic mats and thrombolite cores.** Observed carbonate δ^13^C_CaCO3_ values (blue box) are higher than the maximum theoretical equilibrium δ^13^C_CaCO3_ value (red box). The maximum theoretical equilibrium δ^13^C_CaCO3_ value was calculated from the highest observed δ^13^C_DIC_ value (yellow box) measured among groundwater and lake water samples.

To assess potential metabolic influences on carbonate precipitation in Lake Clifton, the carbon isotope composition of thrombolite calcium carbonate (aragonite) samples was measured and compared to theoretical values in equilibrium with the HCO_3_^-^ in the lake water. Theoretical δ^13^C_CaCO3_ values for aragonite precipitating in equilibrium with HCO_3_^-^ were calculated using the aragonite-HCO_3_^-^ fractionation factor reported by Romanek et al. (1992). Values of δ^13^C_HCO3-_ were calculated from measured δ^13^C_DIC_ values by speciating DIC according to temperature and pH and then applying mass balance constraints assuming carbon isotope equilibrium among DIC species (Deines et al., 1974; Mook et al., 1974). Equilibrium fractionation (over the temperature range 10–40°C) results in aragonite δ^13^C values that are on average 2.7‰ higher than bicarbonate δ^13^C values (Romanek et al., 1992). Therefore, we used the maximum calculated δ^13^C_HCO3-_ value in our dataset to estimate the maximum possible equilibrium δ^13^C_CaCO3_ value. The δ^13^C_DIC_ values of lake water and groundwater samples ranged from -4.30 to 1.55‰ and -13.35 to -7.97‰, respectively. The highest δ^13^C_HCO3-_ value was calculated from the maximum measured δ^13^C_DIC_ value of 1.55‰ and resulted in a maximum theoretical equilibrium δ^13^C_CaCO3_ value of 5.49‰. The lowest δ^13^C_CaCO3_ value measured in thrombolite core samples was 9.02‰ (range 9.02–12.31‰, 1σ = 0.99‰, *n* = 18). Observed carbonate δ^13^C values are therefore 3.5–6.8‰ higher than the maximum theoretical equilibrium δ^13^C_rmCaCO3_ value (**Figure 7**). Groundwater and carbonate δ^13^C values reported in this study are consistent with previously reported Lake Clifton values ranging from -11.7 to -11.8‰ (groundwater) and 4.57 to 10.56‰ (CaCO_3_), whereas the lake water δ^13^C values observed in this study are elevated compared to previous measurements of -7.0 and -7.3‰ (Moore, 1993; Moore and Burne, 1994). The elevated lake δ^13^C values may reflect differences in sampling regimes (i.e., seasonal timing, location) and/or changing hydrological conditions related to the salinity increase, specifically with regard to the source and quantity of groundwater input to Lake Clifton (Warden, 2016).

Measured δ^13^C_CaCO3_ values from Lake Clifton thrombolite cores are markedly higher than theoretical equilibrium δ^13^C_CaCO3_ values, which suggest a photosynthetic influence on calcium carbonate precipitation. Preferential uptake of ^12^C by photosynthetic microorganisms may leave the local DIC reservoir enriched in ^13^C if the rate of DIC supply to the local reservoir is exceeded by the rate of photosynthetic uptake. Carbonate precipitated within this microenvironment (i.e., the microbial mats) would therefore be enriched compared to bulk δ^13^C_DIC_ measurements from the lake or groundwater. Our results are consistent with previous isotope-based interpretations of photosynthetic biosignatures in Lake Clifton thrombolites (Moore and Burne, 1994) but expand on this work by comparing measured results to theoretical equilibrium values and accounting for DIC speciation during data analysis. Photosynthetic activity has also been inferred as the explanation for δ^13^C_CaCO3_ values elevated by 4–5‰ in freshwater microbialites from Kelly Lake (Ferris et al., 1997) and by 1–4‰ in freshwater microbialites from Pavilion Lake (Brady et al., 2010) compared to bulk δ^13^C_DIC_ values.

### Current status of Lake Clifton Thrombolites

Total dissolved solids in Lake Clifton measured 15–32 g/L before a progressive increase in salinity, which began in the mid to late 1990s and may be due to decreases in rainfall and increases in evaporation (Moore, 1987; Rosen et al., 1996; Knott et al., 2003; Smith et al., 2010). The first studies describing the thrombolites were conducted prior to the salinity increase and the documentation of microbialites forming in a hyposaline environment was a significant advance in understanding the broad range of environments in which microbialites can form (Moore et al., 1984; Moore, 1987, 1991; Moore and Burne, 1994). However, recent TDS concentrations in the northern lake basin range from 59 g/L during the winter (2012), when the lake is more dilute, up to 92 g/L during the summer (2014), when evaporative concentration occurs (Warden et al., 2013; Warden, 2016). The extensive and rapid geochemical changes observed in Lake Clifton and apparent reduction in present-day thrombolite growth suggests this ecosystem is under threat of survival, and is therefore of crucial importance to monitor and determine how lithifying microbial ecosystems respond to anthropogenic perturbations in the environment. Although microbialites have had a long fossil record and have adapted to a wide range of environmental conditions (Grotzinger and Knoll, 1999), the Lake Clifton thrombolites provide a unique habitat to examine how microbialite ecosystems respond to rapid climate change events and may provide key insight into the long-term impact of eutrophication and environmental change on microbialite-forming microbial communities.

Despite these dramatic changes, enrichments for genes associated with photosynthesis, CCM, and bicarbonate transport in mats coupled with stable isotopic data associated with the thrombolites suggest that contemporary thrombolite-associated mat communities can control microenvironmental conditions to induce carbonate precipitation, which supports previous studies showing cyanobacteria to be key drivers of calcification in Lake Clifton microbialites (Moore, 1987, 1991). Although, earlier work using microscopic analyses suggested filamentous cyanobacteria (e.g., *Scytonema* sp.) to dominate these systems (Moore and Burne, 1994) our results suggest there has been a dramatic shift in the cyanobacterial population toward coccoid, non-heterocystous forming taxa primarily from the order Chroococcales. The dominance of coccoid cyanobacteria are observed in microbialites of other hypersaline environments such as Hamelin Pool in Shark Bay, Western Australia (Suosaari et al., 2016) and in marine systems, such as Little Darby Island, The Bahamas (Casaburi et al., 2016). However, it should be acknowledged that the currently available reference genomes are skewed toward model organisms such as coccoid *Cyanothece* spp., *Synechococcus* spp., and *Prochlorococcus* spp. as well as heterocystous-forming *Nostoc* spp. and *Anabaena* spp., and this shift in taxa may partially reflect the lack of diversity in sequenced cyanobacterial genomes.

Although our results suggest extensive taxonomic shifts in the thrombolite-associated mats when compared to prior studies, recent microbiome studies of microbialite ecosystems targeting other environmental systems have shown that taxonomic composition is not as critical to inducing carbonate precipitation as the overall metabolic capabilities of the system (Zhang et al., 2015; Casaburi et al., 2016). Additionally, the presence of genes associated with osmotic stress in both thrombolite- and sediment-associated mats suggest that the Lake Clifton microbial community is adapting to the higher salinity observed in the lake. These differences could also reflect the seasonal variations in water levels as lithifying mat systems have been shown to rapidly shift in microbial composition due to changes in salinity and nutrient levels (Dupraz et al., 2013). As Lake Clifton represents one of few known thrombolite-forming ecosystems, it is imperative to continue monitoring this at-risk environment. Observations in February and March 2014 suggest that thrombolite-associated microbial mats are primarily limited to the northeastern foreshore of Lake Clifton and that thrombolites outside of this area are mostly inactive. Future work comparing active and inactive areas will be critical to more fully elucidate the environmental constraints on mat growth and thrombolite accretion in this threatened ecosystem.

## Author Contributions

JW led field activities including the sample collection, conducted the experiments for the project, analyzed data, and wrote the initial draft of the manuscript. GC assisted in the development of the analysis pipeline for the molecular data. DB assisted with fieldwork and with stable isotope data analysis, PB assisted with fieldwork and geochemical interpretation, and CO assisted with fieldwork and interpretation of molecular data related to microbial activity. JF assisted in molecular data analysis. All authors assisted in the writing and editing of the manuscript.

## Conflict of Interest Statement

The authors declare that the research was conducted in the absence of any commercial or financial relationships that could be construed as a potential conflict of interest.
