# Supplementary material for: Characterization of Microbial Mat Microbiomes in the Modern Thrombolite Ecosystem of Lake Clifton, Western Australia Using Shotgun Metagenomics
Source: Front Microbiol. 2016 Jul 6;7:1064. doi: 10.3389/fmicb.2016.01064 (PMC4933708; doi:10.3389/fmicb.2016.01064)
Supplement: Supplementary file 5 [file Supplemental_Figures.PDF]

Supplemental Material

Characterization of microbial mat microbiomes in the modern thrombolite ecosystem of Lake Clifton, Western Australia using shotgun metagenomics

John G. Warden[1,2], Giorgio Casaburi[2], Christopher R. Omelon[1], Philip C. Bennett[1], Daniel O. Breecker[1], Jamie S. Foster[2*]

[1]Department of Geological Sciences, University of Texas at Austin, Austin, TX, USA

[2]Department of Microbiology and Cell Science, University of Florida, Space Life Science Lab, Merritt Island, FL, USA.

Running Title: Living thrombolites of Lake Clifton

***Corresponding Author**

Email: jfoster@ufl.edu

Key words: thrombolite, microbialite, stable isotope, Lake Clifton, microbial mat

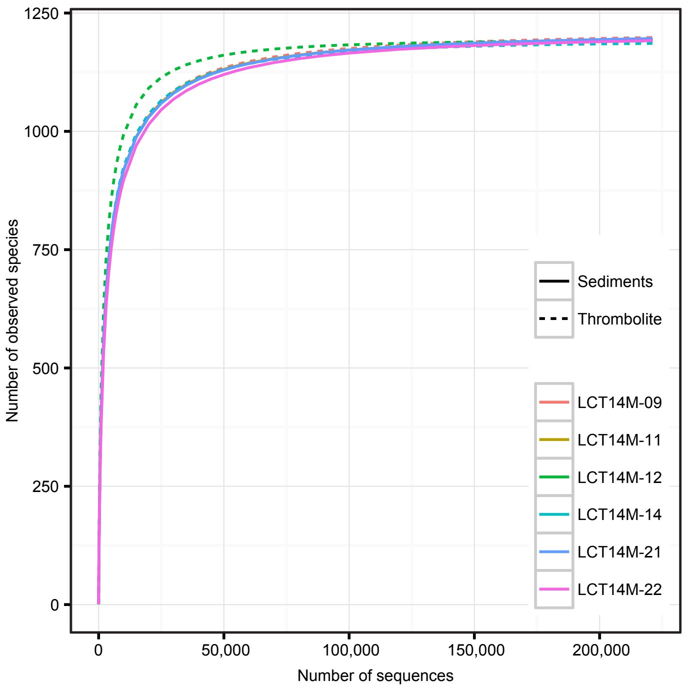

FIGURE S1. Rarefaction curves according to different mat types and location. Rarefaction curves for each individual sample were generated as computed by observed species at a depth of 221,168 sequences/sample. Bacterial community richness did not differ between sediments and thrombolites ($p > 0.05$). Every sample in the study showed similar trend in the number of observed species in relation to multiple sequencing depths.

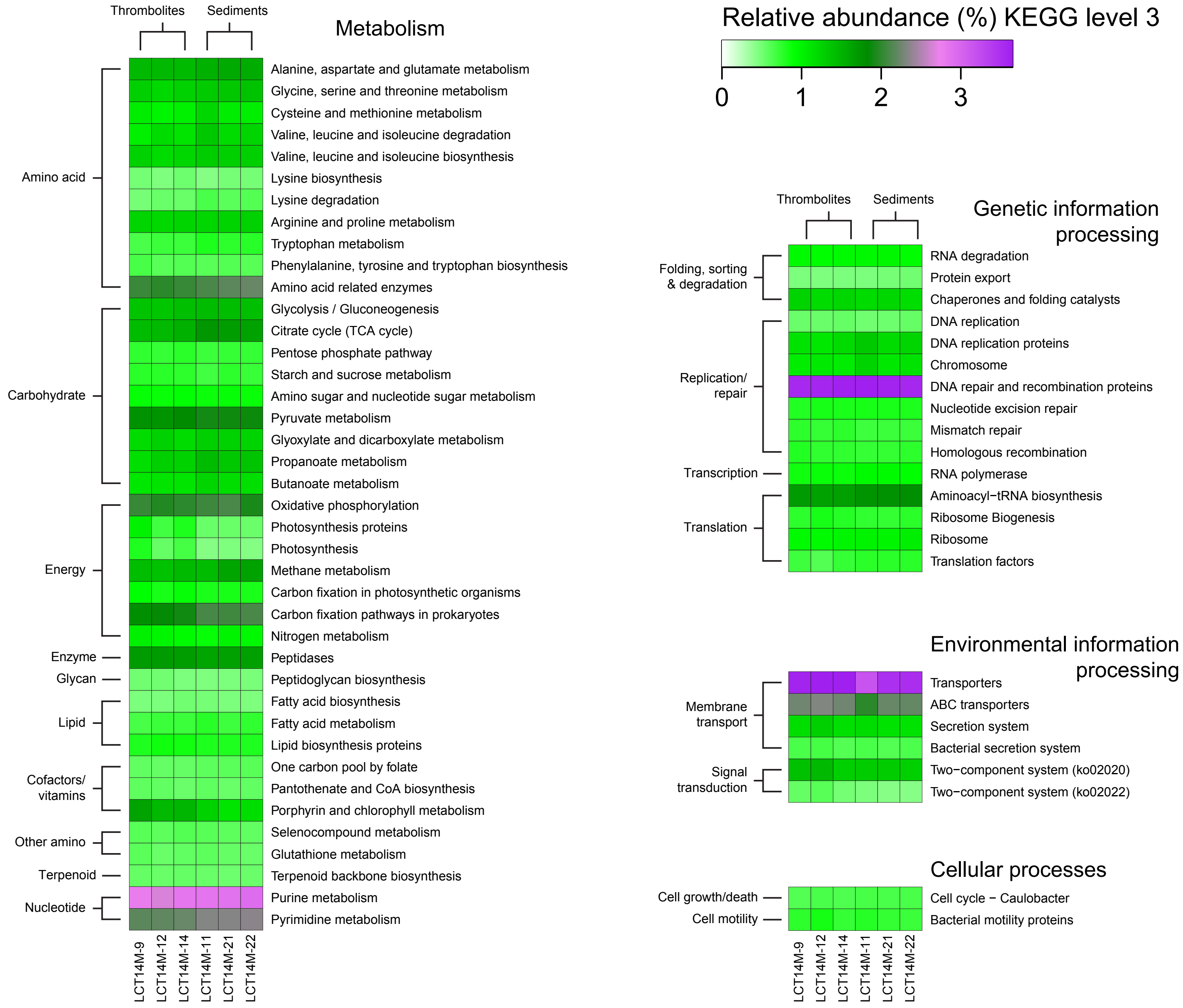

FIGURE S2. Relative abundance of level 3 KEGG pathways in sample metagenomic libraries. Heatmaps show level 3 KEGG pathways present in sample metagenomes at a relative abundance of greater than 0.5%.

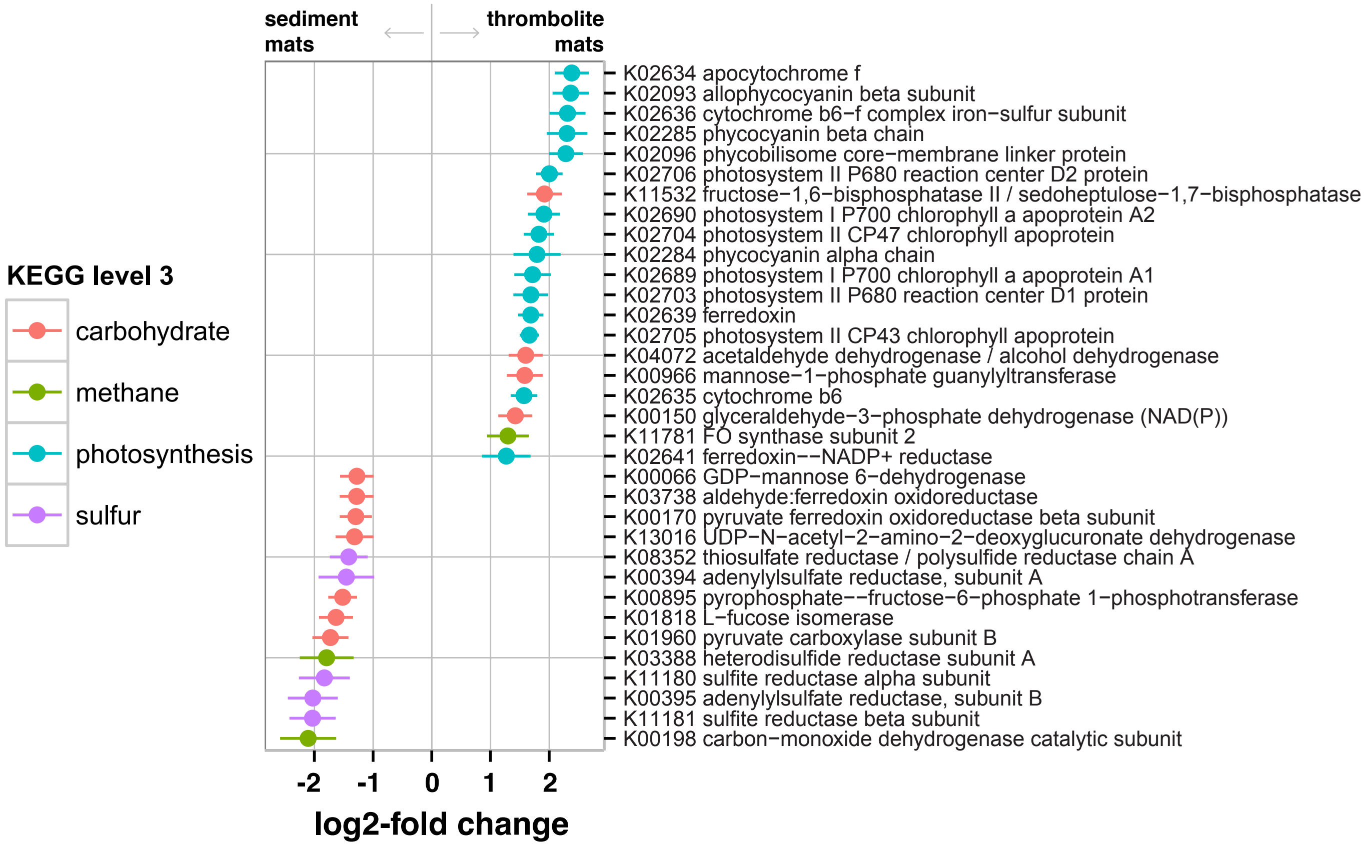

FIGURE S3. Selected level 4 KEGG metabolic functions that are significantly enriched in thrombolite- and sediment-associated mats. A positive log2-fold change indicates genes significantly enriched (Wald test, $p<0.05$) in thrombolite-associated mats and a negative log2-fold change indicates genes significantly enriched in sediment-associated mats. Colors indicate the level 3 KEGG grouping for each data point and lines represent standard error. Results are reported for photosynthesis, carbohydrate, methane, and sulfur metabolisms.

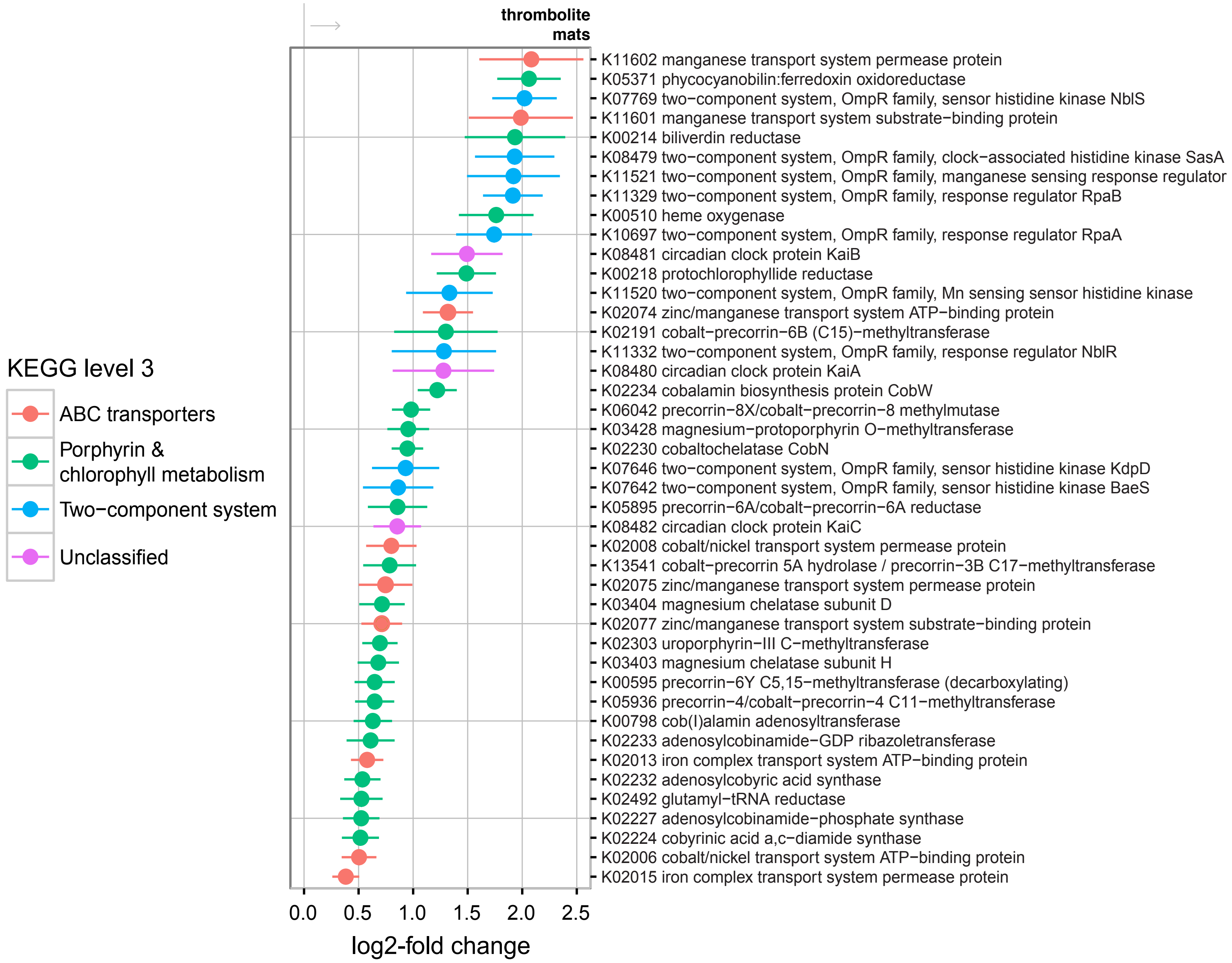

FIGURE S4. Selected level 4 KEGG metabolic functions that are significantly enriched (Wald test, p<0.05 in thrombolite-associated mats. Colors indicate the level 3 KEGG grouping for each data point and lines represent standard error. Results are reported for selected functions within ABC transporters, porphyrin and chlorophyll metabolism, and two-component system.

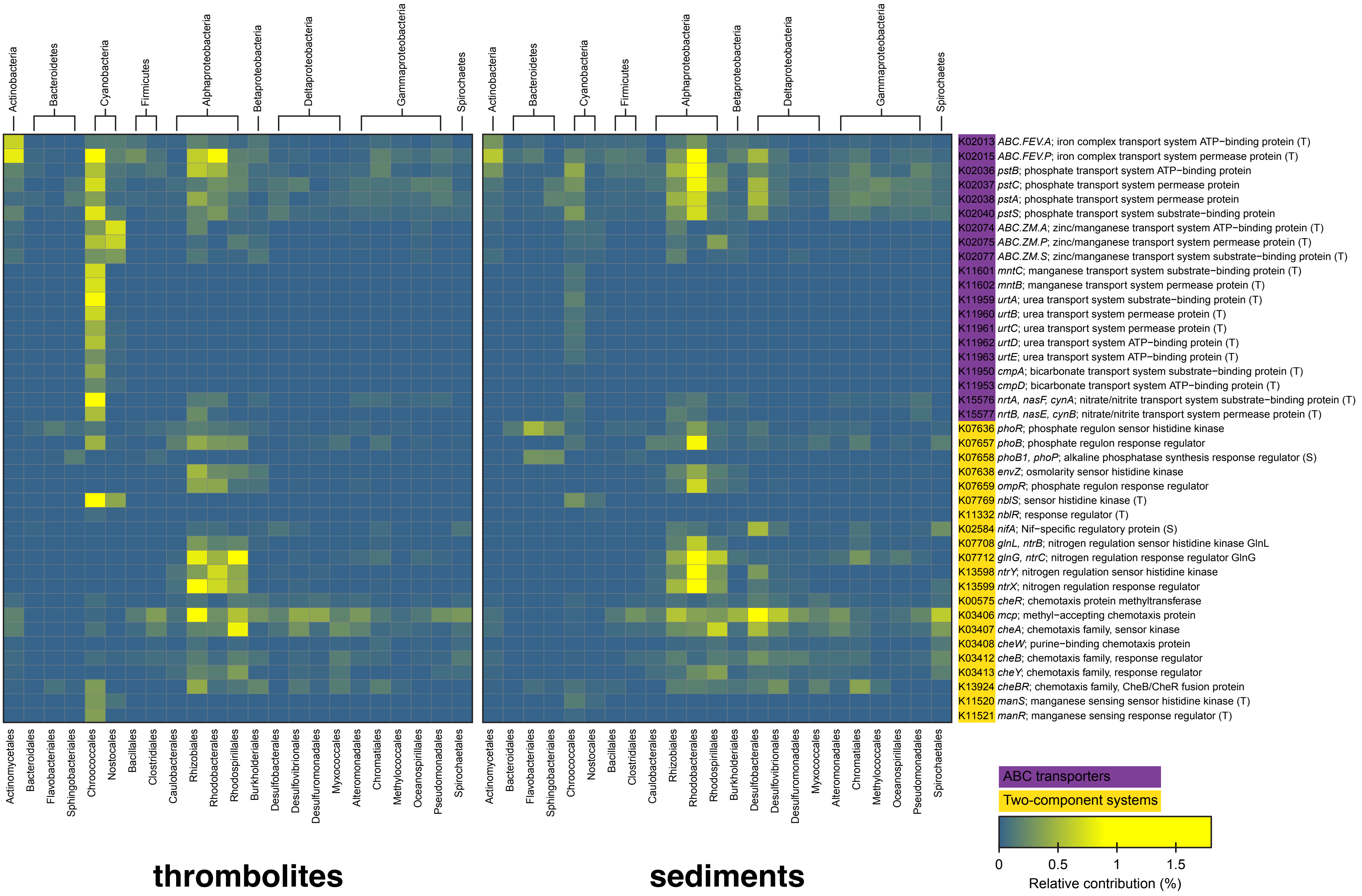

FIGURE S5. Bacterial taxa associated with selected KEGG level 4 functions from the pathways two-component systems and ABC transporters. Heatmaps show the relative contribution of each taxon to the total number of selected genes for t hrombolite- and sediment-associated mats.
